# Editorial: Cytoskeletal alterations in aging and disease

**DOI:** 10.3389/fcell.2023.1359465

**Published:** 2024-01-08

**Authors:** Monika S. Brill, Coralie Fassier, Yuyu Song

**Affiliations:** ^1^ Institute of Neuronal Cell Biology, Technical University Munich, Munich, Germany; ^2^ Munich Cluster of Systems Neurology (SyNergy), Munich, Germany; ^3^ Sorbonne Université, INSERM, CNRS, Institut de la Vision, Paris, France; ^4^ Department of Neurology, Harvard Medical School, Boston, MA, United States; ^5^ Department of Neurology, Massachusetts General Hospital, Boston, MA, United States

**Keywords:** microtubule, neurofilament (NF), microtubule associated proteins, cytoskeleton, actin, aging, disease, posttranslation modification

The cytoskeleton of eukaryotic cells consists of microtubules, actin and intermediate filaments, which are highly interconnected and compartmentalized in polarized cells, such as neurons (Iwanski and Kapitein; Kevenaar and Hoogenraad, 2015; Leterrier et al., 2017). In particular, these filaments are much more than just pure structural elements and ensure various additional functions critical for neuronal development and maintenance (Iwanski and Kapitein; Dent and Baas, 2014; Coles and Bradke, 2015; Yuan et al., 2017). Not surprisingly, dysfunction of any of these components can cause neurodegenerative diseases, such as Alzheimer’s, Charcot-Marie-Tooth disease (CMT) or Hereditary spastic Paraplegia (HSP) (Martnez-Hernandez et al.; Morris and Brady; Bomont et al.; Costa and Sousa; Matamoros and Baas, 2016).

Microtubules, which are rod-like, polarized structures assembled from alpha/beta-tubulin dimers form the basis for organelle transport and modulate cell shape and behavior (Conde and Caceres, 2009; Guedes-Dias and Holzbaur, 2019). Interestingly, microtubules can be highly heterogeneous across cellular and subcellular compartments due to several factors: 1) cell-type specific expression of tubulin genes; 2) locally active enzymes catalyzing posttranslational modifications (PTMs); and 3) various flavors of Microtubule-Associated Proteins (MAPs) (Janke and Bulinski, 2011; Roll-Mecak, 2020; McKenna et al., 2023). However, how these different factors assemble and segregate in developing and mature neurons to drive specialized microtubule functions remains elusive (Chakraborti et al., 2016; Bodakuntla et al., 2021; Moutin et al., 2021; Atkins et al., 2023; Pero et al., 2023).

Recent innovative strategies to label or image specific microtubule subpopulations with high resolution led to novel concepts suggesting that the tubulin and MAP codes can cooperatively and locally drive microtubule lattice compaction or expansion, renewal, and rescue to tune microtubule structure and dynamics (Iwanski and Kapitein; Janke and Magiera, 2020). In addition, all cytoskeleton elements and associated proteins are heavily modified through PTMs, many of which are associated with neuronal development and degeneration. However, key questions remain: Do PTMs and MAPs have functional roles in driving the neuronal states, or are they simply indicators to reflect signaling changes associated with these different states, or both? To start answering these questions, three articles in this Research Topic illustrate the role of tubulin PTM crosstalk in AD progression (Martinez-Hernandez et al.), the impact of a major AD-related MAP, Tau, and its phosphorylation on axonal growth (Morris and Brady), and the effect of neurofilaments and their PTMs in neuronal homeostasis and neurodegenerative diseases (Kotaich et al.).

Martnez-Hernandez et al. showed that PTMs, such as tubulin acetylation and tyrosination/detyrosination, influenced each other and could affect AD progression by altering microtubule dynamics. Morris and Brady showed that site-specific phosphorylation of tau may lead to tau conformational changes beneficial for normal neurite development. Both papers illustrated the unconventional roles of cytoskeletal PTMs that depend on neuronal states: tubulin PTMs, which are critical in normal development, can exacerbate AD progression when misregulated, while selective tau phosphorylation, which is indicated in AD, may promote axonal growth when physiologically regulated. These intriguing results are important for understanding neuronal cytoskeleton in health, disease and aging through multiple lenses focusing on different neuronal states. However, the underlying molecular mechanisms require further studies so that we may know how to regulate these PTMs, which can be a double-edged sword for neurons. In this context, the review by Kotaich et al. pointed out that the dynamicity of neurofilaments, which is based on their fine-tuned assembly/transport/degradation to sustain key structural and electrophysiological properties of the neurons, is critically influenced by PTMs (Yuan et al., 2017; Yuan and Nixon, 2023). These data shed new perspectives on the etiology and treatment of neurofilament-related neurodegenerative disorders (e.g., CMT or amyotrophic lateral sclerosis, (Rao and Nixon, 2003; Perrot and Eyer, 2009; Didonna and Opal, 2019; Laura et al., 2019; Stone et al., 2021).

Importantly, neurofilaments were shown to influence microtubule dynamics in neurons (Yadav et al., 2016). Such crosstalk may be established and regulated by PTMs and MAPs, leading to fine-tuning of neuronal morphology, cytoarchitecture, and physiology. This proposed mechanism may provide an additional step in untangling the complexity behind the cytoskeleton-mediated regulation of neuronal homeostasis and its contribution to aging and diseases. Following this idea, determining how cytoskeleton composition and axon morphology change during aging would be very informative (Kounakis and Tavernarakis, 2019; Kim et al., 2022). Interestingly, by examining sensory axons from healthy human skin biopsies, Metzner et al. reported increases in cytoskeleton composition in both sexes and larger axonal caliber in male during aging. They proposed that such changes may modify axonal function, thus, contributing to aging-related decrease in sensory perception or increased susceptibility to degeneration.

Another emerging theme from several articles concerns the key physiological roles of cytoskeleton-associated proteins incriminated in neurodegeneration or other aging-related disorders, such as the microtubule-regulatory proteins Gigaxonin (peripheral neuropathies; Kotaich et al.; Arribat et al., 2019), Tau (AD; Morris and Brady; Li et al., 2014), Spastin (HSP; Costa and Sousa; Yu et al., 2008; Brill et al., 2016; Jardin et al., 2018), or the F-actin binding protein Radaxin (Hearing loss; Hausrat et al.; Paglini et al., 1998) during neuronal development. This is particularly emphasized in the review from Costa and Sousa, summarizing the numerous studies demonstrating that tight control of microtubule dynamics and membrane trafficking by Spastin determined several key steps in neuronal circuit wiring (e.g., axonal outgrowth/branching, synapse elimination/maintenance). The authors further highlighted that fine tuning of Spastin expression and activity through post-transcriptional/-translational modifications and MAPs, is fundamental for both axonal development and homeostasis.

Altogether, these studies suggest cytoskeleton alterations as a major continuum between neuronal circuit development and dysfunction/degeneration and left us with open questions: How do mutations or PTMs in cytoskeleton-associated proteins with key developmental functions lead to late-onset neurodegeneration or aging-related disorders? Does a developmental component exist for these diseases? And if not, what molecular mechanisms compensate for their dysfunctions in neuronal development, and how are they lost during aging? Answering these questions will deepen our understanding of fundamental neurobiology and generate innovative therapies for neurodegenerative and other aging-related diseases.
